# The Relationship between Inequalities in Household Sports Consumption Expenditures and Income Level

**DOI:** 10.3390/ijerph192315608

**Published:** 2022-11-24

**Authors:** Dávid Paár, Zoltán Pogátsa, Pongrác Ács, András Szentei

**Affiliations:** 1 Faculty of Health Sciences, Institute of Physiotherapy and Sport Sciences, University of Pécs, H-7621 Pecs, Hungary; 2 Alexandre Lámfalussy Faculty of Economics, University of Sopron, H-9400 Sopron, Hungary; 3 Faculty of Health Sciences, Doctoral School of Health Sciences, University of Pécs, H-7621 Pecs, Hungary

**Keywords:** households, income, sports expenditure, sports consumption, inequality

## Abstract

Inequalities in income, wealth, quality of life, health and education are an intensively researched field of economics. In this study, we examine the inequality in sports expenditures of Hungarian households. We hypothesize that the development of income inequalities will also correlate significantly to inequalities in sports consumption, and this trend has been intensifying over the past two decades. The research is based on the Household Budget Survey database of Hungarian households for the period 2005–2017. The net income conditions of the population and the sports expenditure items recorded on the basis of the COICOP nomenclature are examined by income decile. Data is analysed using descriptive statistics, inequality indicators and correlation calculations. Aggregate household expenditures on passive sports consumption show a stagnant trend, while aggregate expenditures on active sports consumption follow a slightly upward trend among the Hungarian population. Inequality indicators show growing inequalities in terms of income and sports expenditure over the reviewed period. Income inequality and sports spending inequality move together. The Hungarian population is becoming polarised in terms of both income and level of sports expenditure.

## 1. Introduction

### 1.1. Inequalities in the Economics Literature

During most of the second half of the twentieth century, the question of social inequalities only featured on the periphery of the study of economics. In 1958, Galbraith could rightfully note that “few things are more evident in modern social theory than the decline of interest in inequality as an economic issue” [1]. The subject was externalized to the discipline of sociology. The issue was brought back into the focus of the economics profession by Piketty [2]. Piketty then expanded his masterpiece to include several centuries and, effectively, the entire globe [3].

A debate about the causes of inequality has also been reignited [4,5]. This research has shown that it is mostly the lack of adequate redistributive mechanisms that is responsible for high levels of inequality, as described by Rawls in his treatise on justice as fairness [6,7]. Inequality can be measured with stock or with flow indicators, such as wealth and income. In addition, we can also study social mobility within a single lifetime, or by using the previous generation as a baseline. Studies show that large inequalities and/or a lack of social mobility can have detrimental consequences for society at large. Higher inequality leads to inferior social outcomes not only for the poor, but also for the more affluent members of the same society [8,9].

Of course, inequality is a highly polarizing subject, inviting much ideological debate. At the other end stands the towering figure of Schumpeter, who famously argued [10] that inequalities are necessary, in that they are the driving force behind capitalism. Without an opportunity to get ahead of others, he argued, people could not be motivated to make the extra effort. In politics, this was taken up by Reagan’s supply-side revolution in the eighties, and in the scientific studies of Forbes [11]. The Schumpeterian thesis has been heavily disputed by other authors, such as Birdsall, Ross and Sabot, who argued that a more egalitarian society is likely to invest more in human resources across society [12]. This is backed up by the phenomenal growth trajectories of egalitarian societies in the Far East, such as Taiwan or South Korea. Famously, Acemoglu and Robinson have theorized this, arguing that successful societies, regardless of professed ideology or political system, shared the common feature that they were inclusive rather than extractive, meaning they spread development resources society-wide, rather than concentrating them in the hands of a narrow elite or an even smaller clan [13].

Important research has also been carried out on global inequalities, as well as comparisons of inequalities across nations [14]. This research has found that inequalities between nations are larger than inequalities within almost any nation on Earth. It has also discovered that the nation one is born into is the single most important determinant of the standard of living of an individual.

### 1.2. Inequalities in Hungary

Inequalities in Soviet style countries were low by definition, forming part of the auto-narrative of the political system. Most of these societies, Hungary included, came out of political and economic transition with low levels of inequality in terms of international comparison.

As Piketty points out, some formerly ‘communist’ societies, such as Russia and China, have since developed very high levels of inequality, comparable to or exceeding that of the United States. This is generally not true of the Central Eastern European region, where inequalities have remained low in terms of international comparison [3].

The history of measuring inequalities in post-transition Hungary is an interesting one. After transition, public discussion about income inequalities slowly faded out. It was generally assumed that capitalism would lead to greater inequalities, but exactly how wide these would become, or indeed how wide they should be allowed to become, was less and less often discussed.

Former Communist economies started out, at around the time of their economic transitions in the late 1980s, with very low income Ginis in terms of global comparison, as it was an ideological goal of their one-party governments to attain a relatively equal society. For methodological reasons, we only have sporadic figures from the time period when these transitions were already under way, and their income inequalities were already likely to have been on the increase.

It is striking how differently the former Communist states ended up after transition. The Visegrad states concluded their economic transitions after 2005, with an income Gini in the range of 0.25–0.35, which is comparable to neighboring Austria and Germany. China ended up in the much higher 0.39–0.44 range, as did Russia. This is much closer, and often even exceeds, the longer-term income Gini trend of the United States, at around 0.40–0.41. The differences between the eventual income inequality levels of these post-communist regions can be explained by their different approaches to economic transition. By comparison, the highest income Gini in a major economy of the world can be found in post-Apartheid South Africa (0.60–0.65 range) [15]. 

Measures of wealth inequality are harder to come by, as Hungary does not have a national level wealth tax. Consequently, there is no reliable and comprehensive wealth register. Researchers find it impossible to compile a consistent and reliable database akin to Piketty’s studies of the United States, Britain, and France. The debate about wealth inequalities was only really reignited by a recent book authored by Éber [16], the title of which describes Hungarian society as being shaped like a ‘water droplet’ in terms of both income and wealth. Éber makes use of data from the Central Bank to claim that most Hungarians are found in the wider bottom half of this globule, while over 50% of national wealth is owned by a very narrow elite in the upper ‘stem’ of the elongated droplet. This poses a very complex challenge. Wealth inequalities experienced first-hand in everyday life are low since, due to their position in the bottom globule, the majority of people only personally know people who are relatively close to them in terms of social status. A few decades ago, this would have been the defining feature of people’s awareness of their social position. However, nowadays the media regularly reports on the lifestyle of the super-rich narrow elite, which means citizens lower down are well aware of the extremely concentrated wealth of this top part of the distribution. Thus, paradoxically, it is fair to say that wealth inequalities are simultaneously low and high in Hungary.

One way to capture this apparent anomaly would be to use the so-called Palma ratio instead of the more widely used Gini, developed by Cambridge economist Palma. This consists of the ratio of the income or wealth share of the top decile to that of the bottom two quintiles. It is based on the observation that changing inequality is substantially a shift from the poorest 40% to the top 10%, and vice versa. A similar approach is taken by leading inequality researchers Pikkety, Saez and Atkinson, who often look at the income and wealth ratios of the top 10%, the top 1% and the top 0.1%. The key problem in the case of Hungary, however, is that, due to a lack of a nationwide property tax, there is no reliable data available on the wealth of the top 10% of the 1%.

**Figure 1:**
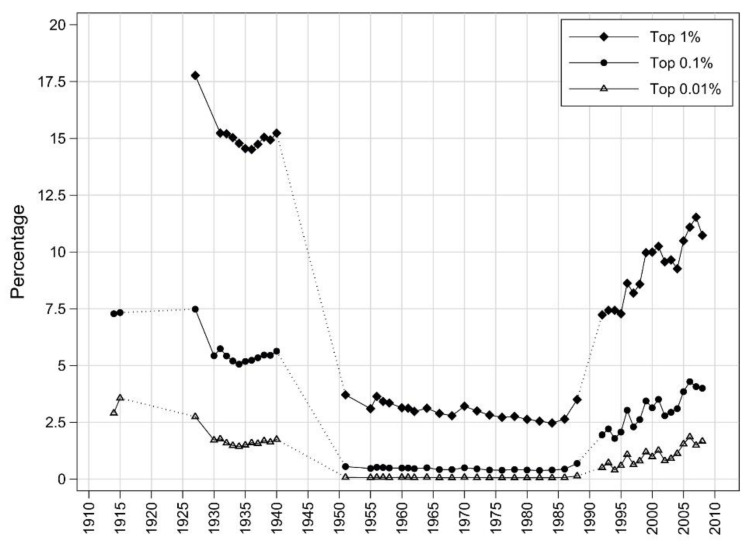
The income share of the top 1%, 0.1% and 0.01% has increased considerably after transition to capitalism in Hungary [17].

Mavridis and Mosberger observe a dynamic rise in the income share of the top 1%, 0.1% and 0.01% in Hungary after transition. Even though Hungary’s income Gini is low in terms of international comparison, the share of the top 1% in Hungary is higher than in China, and comparable to India, two very unequal societies (Figure 1) [17].

It is social mobility that is the key problem in the case of Hungary. Although domestic studies have almost disappeared in this respect in the new millennium, international studies have indicated that Hungarian society is by and large frozen. Key in this respect is a Europe wide international comparison, based on data collected between 2007 and 2010 [18]. This research has found Hungary to have the lowest levels of social mobility in the entire European Union, both in the case of men and women.

There have been no comprehensive studies on social mobility since the Eurofound study [18] at the national or the EU level. However, since government expenditure on the most crucial social mobility enhancing subsystems (education, healthcare, social policy, etc.) have been reduced since, as a percentage of GDP, it is reasonable to anticipate that social mobility has in fact declined even further.

### 1.3. Inequalities and Sport Consumption

Several states with lower income inequality outperform richer countries with higher inequality regarding various welfare, health and social measures [9]. Higher wealth inequality threatens the stability of industrial economies [3,19]. Among the global risk factors, we find “serious income inequalities” [20]. Increase in income inequality may lead to a rise in consumption inequality [21]. A high correlation has been demonstrated between leisure time and income, which was negative in the cases of Norway, Finland, Belgium, Germany, France and the United Kingdom and positive correlation in the cases of Sweden, Slovenia, Spain, Estonia and Poland. It was also examined by multiple regression analyses that GDP per capita and leisure time is correlated [22]. Income inequalities and GDP per capita have shown significant differences in relation to participation in sport and recreation activities [22]. A one percentage point increase in income inequality has a significant impact (a 40,008 USD increase) in annual healthcare costs in the United States [23]. The value of the Gini coefficient has been demonstrated to show negative correlation with life expectancy and other health indicators such as high blood pressure, depression or BMI [24,25,26]. Active participation in sports has been shown to have a strong relationship with physical and mental health, which directly affects discretional income, and lowers health costs [27]. In the literature on sports consumption, the Gini index, S80/S20, P90/P50, and GDP/capita are frequent indices for income inequalities [22,28].

### 1.4. Passive Sport Consumption

Changes in income and qualifications have a positive impact on participation in both amateur and professional sport events [29]. Among the highest income group, 54% of men and 32% of women participate in sports events in Canada. For the lowest income group, this number decreases to 13% of men and 10% of women [29]. The connection between household income and the participation rate in amateur sport events was positive and linear in the United States based on the NORC General Social Survey data, which was based on representative sample of Americans [30]. As for the entire European Union, based on the EU SILC database, average passive participation in 2015 in events showed that 71% did not participate at all, 17% participated 1–3 times, and 12% at least 4 times. The highest rate of activity was found in the Netherlands (54%), the lowest in Romania (17%). Hungary (25%) was placed in the lower end in terms of passive participation [31]. 

### 1.5. Active Sport Consumption

**Figure 2:**
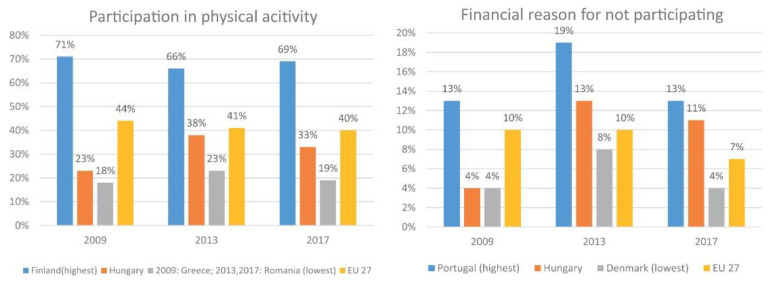
Participation in physical activity at least once a week or more and financial burden of physical inactivity in the EU [32,33,34].

**Figure 3:**
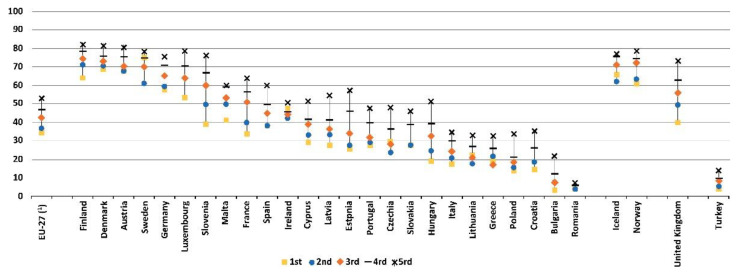
Practicing sport, fitness or recreational (leisure) physical activities at least once a week, by income quintile, 2014 [31].

There has been a decreasing trend in active sports participation since 2009 in the EU countries [32]. In terms of active participation in sports at least once a week or more, the most active country was Finland (69%), the least active Romania (40%) in 2017. Hungary (33%) is found in the lower end, with a slight increase in activity numbers (Figure 2) [32,33,34]. More developed EU countries do not only have higher per capita GDP figures on a purchasing power parity comparison, but their physical activity and sport expenditure levels are also higher [27,35,36]. Paár clustered European countries by four attributes (sports consumption, mortality, Gini coefficient, average consumption). The results showed that the countries in the best positioned cluster had highest sports related expenditure, lowest income inequality, high average consumption and high life expectancy (e.g., Scandinavian countries). Hungary was found to be in the worst positioned cluster, along with other East Central European countries, with the lowest sports related expenditure, average income inequality, lowest average consumption and shortest life expectancy [35]. Tudor-Locke et al. examined daily steps and their connection with individual factors: daily steps were proved to be a significant factor among the top and the bottom income groups [37]. Among EU respondents based on the regularly recorded Eurobarometer studies, the most important reason for not participating more regularly in physical activity was financial. Portugal (13%) showed the highest numbers, Hungary increased from 4 to 11% from 2009 to 2017, and Denmark (4%) showed the lowest (Figure 2) [32,33,34]. In 2013, amongst EU respondents who participated in sports or physical activity at least once a week, Slovenia showed the biggest gap between the top and the bottom income quintile groups (37 points). Hungary followed in second place (33 points) and Ireland, Romania and Sweden had the lowest gaps (2–3 points) (Figure 3) [34]. In recent years, income inequality and physical activity guidelines were examined: among women in the United States income inequalities are associated with higher odds of non-participation in physical activity, and not meeting the recommendations for physical activity guidelines [38].

### 1.6. Aim of the Research

The aim of this research is to examine the development of income inequality and sport expenditure inequality and their relationship over the past two decades in Hungary using both longitudinal and cross-sectional data. We hypothesize that there is a relationship between the two phenomena, as income status is one of the main determinants of the level of sports expenditure [39,40,41]. If there is a relationship between the two phenomena, it may be worthwhile to investigate in more depth the influence of the background variables that drive the increase in inequalities.

## 2. Materials and Methods

The database forming the basis of the analysis was the annual Household Budget Survey (HBS) collected by the Hungarian Central Statistical Office (HCSO), based on a stratified, multi-stage sampling methodology, in the 2005–2017 period. HBSs are nationally representative with regards to Hungarian private households. The sample sizes for each year are summarized in Appendix A, Table A1. The database contains weights for each responding household, which indicate how many other households are represented nationally by the responding household. This makes it possible to aggregate specific income and expenditure categories.

HBSs use the Classification of Individual Consumption by Purpose (COICOP) nomenclature, based on Eurostat methodological recommendations. There was a methodological change in data collection in 2015, which limits time series comparisons of sports expenditure items, but not inequality indicators. Our research makes use of the income and sports expenditure items (related to both active and passive sports consumption) listed in Table 1. Sports expenditures were aggregated into an aggregate indicator. Table 1 also contains the consumer price indices used by the CSO to convert these items to inflation adjusted value in 2017.

The household level data was calculated per capita, using household size, as also recorded by the HBS. Income deciles determined on the basis of net income per capita were used to analyze inequalities.

The weighted net incomes and sports expenditure items of households was aggregated. Averages for the total sample size, as well as for income deciles per each year, were also calculated. In addition to the full sample database, the averages were calculated separately for a sub-sample of households for sports expenditures.

The following indicators based on the share of each income decile in the total sample were calculated to demonstrate inequalities:

- Gini coefficient;
- normalized Herfindahl-Hirschmann Index;
- Robin-Hood Index;
- ratio of the highest and lowest income decile aggregate values (Q10);
- ratio of the highest and lowest income quintile aggregate values (Q5).

The relationship between income and sports expenditure inequalities was examined using the correlation coefficient.

The cross-sectional data is presented for the first year of the time series (2005) and for the years 2009, 2013, and 2017, in which years the representative Eurobarometer surveys on non-professional sports behaviour were carried out [32,33,34].

## 3. Results

### 3.1. Aggregate Income and Sports Expenditure Data

**Figure 4:**
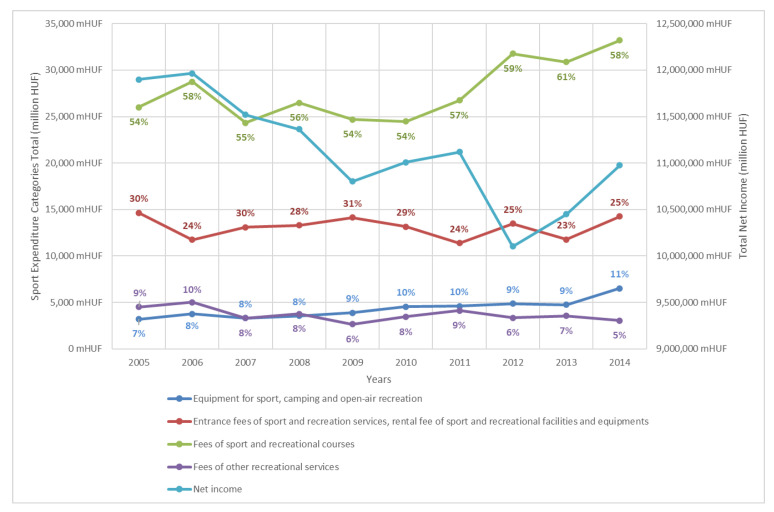
Development of sport expenditure’s aggregate values and incomes between 2005 and 2014, (percentage data shows the proportion of aggregated sport expenditure items). Source: own calculation.

The development between 2005 and 2014 of aggregate data and its relative weight is shown in Figure 4. (Appendix A, Table A2 contains the data between 2015 and 2017. However, comparison is impossible because of changes in collection methodology.) The low point of household incomes was in 2012, with an increasing trend after that. Sports expenditure data shows stagnation in this period, except for a slight increase in “fees for sports and recreational courses”. This most significant category accounts for more than half of all sports expenditure each year. The second largest category is “entrance fees for sports and recreational services, rental fees for sports and recreational facilities and equipment”, with a 23–31% share. Other categories such as “equipment for sports, camping and open-air recreation” and “fees for other recreational services” had a significantly lower share.

### 3.2. Proportion of Population with Sports Expenditure

**Figure 5:**
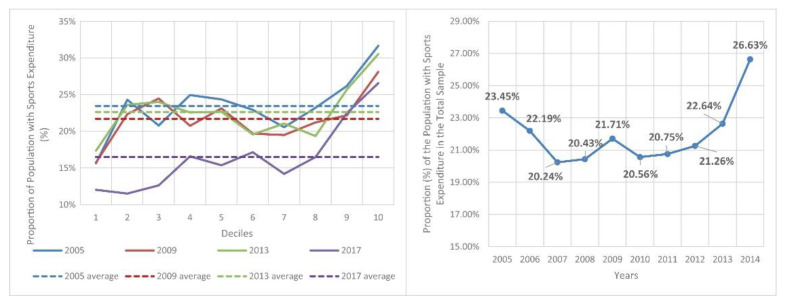
Proportion of population with sports expenditure (%). Source: own calculation (continuous legends: proportions in the given deciles, dotted legends: proportion in the whole populations).

The proportion of the population with sports expenditure, per deciles and in total each year, is summarized in Figure 5. This proportion is much higher in the two uppermost deciles, while it is much lower in the first one than in the sample average. The other deciles are scattered around the sample mean. (Both the average numbers of households with sports expenditures and without sports expenditures are highest in the first decile and decrease continuously up to the top decile. The average number of households with sports expenditures is higher than in households without sports expenditures in each year and in every decile.) The total sample average shows a decreasing trend between 2005 and 2007 and an increasing one since 2007.

### 3.3. Income and Sports Expenditure per Capita 

**Figure 6:**
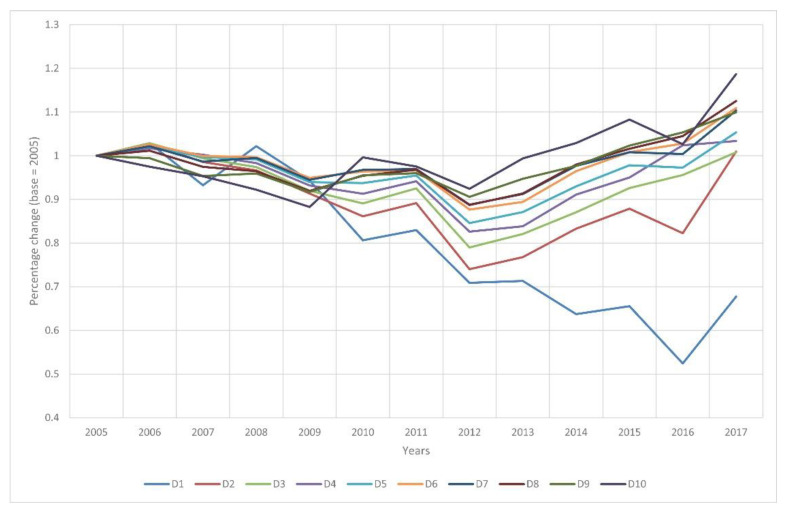
Average annual per capita income of each income decile compared to 2005 between 2005 and 2017. Source: own source.

Figure 6 shows the development of per capita income, compared to the year 2005, for the period 2005–2017. There is a decrease for every decile after 2005, but the extent is varied. In the case of the lower deciles, the decline lasted longer and was more significant. The incomes started to increase again from 2012 onwards for most deciles. The uppermost decile was able to return to the 2005 level by 2013, and the other deciles by 2015, 2016 or 2017, except for the lowest decile. By the end of 2017, the top decile was able to increase its average income to 118.69%, while the lowest decile was still only 67.73% of its 2005 value.

The average of net income per capita was also calculated for the total sample and the sample of households with sports expenditures in each decile. There is a gap between households with sports expenditures and the overall sample, in the case of the first and the tenth deciles, as well as the total sample. Households with sports expenditures had a higher average income in the bottom decile in 2009 and 2013, while it was lower by more than 70,000 Hungarian Forints (HUF) in 2017. The average income of households in the top decile and in the total sample with sports expenditures was higher in each year (Table 2).

**Figure A1:**
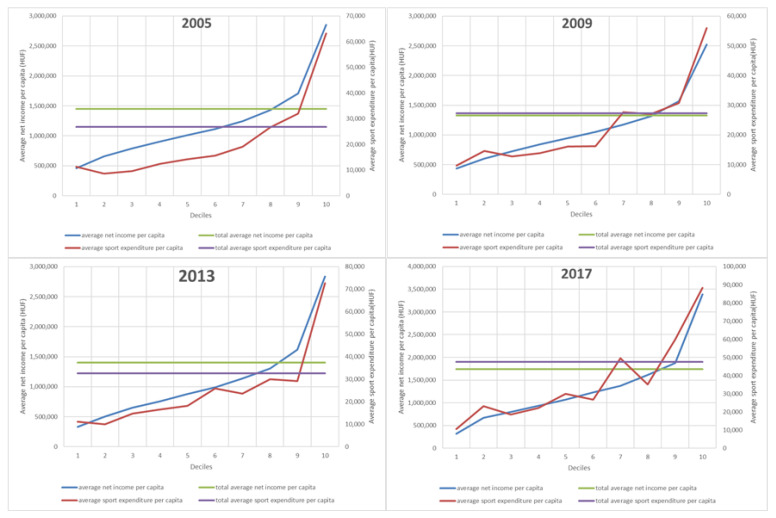
Average net income per capita and sports expenditure per decile in the sub-sample of people with sports expenditures (HUF). Source: own calculation.

There is a significant difference in per capita sports expenditure between the average values of the total sample and households with sports expenditure, because almost 80% of the overall population does not have any sports expenditure. Moving from the bottom decile towards the top, there is an almost continuous increase in sports expenditure. The growth rate becomes exponential for the top two deciles. Only the top two deciles have sports spending above the average of the total sample. (Table 3, Appendix A, Figure A1).

**Figure 7:**
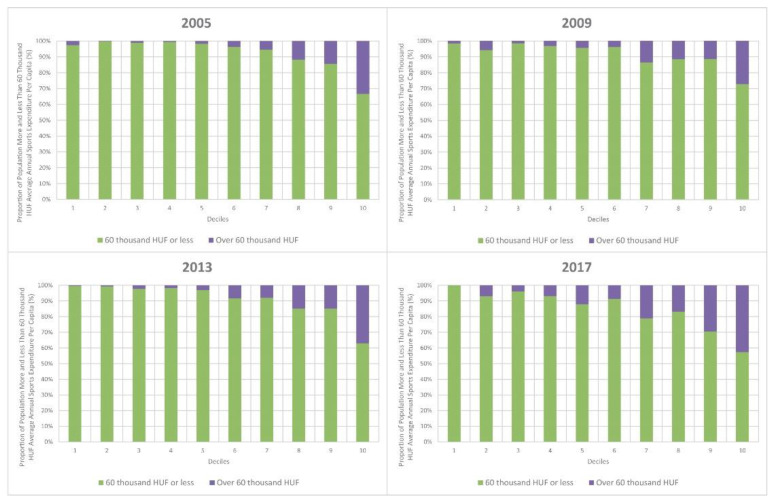
Proportion of population more and less than 60,000 HUF average annual sports expenditure per capita (%). Source: own calculation.

Choosing as a benchmark an annual sports spending of 60,000 HUF per capita—roughly equal to two first-class sports matches per month or a monthly gym ticket for half a year—we examined the share of the population with at least such items of sports expenditure in each decile. Within the sub-sample of households with sports expenditures, 10.80% in 2005, 10.90% in 2009, 13.80% in 2013 and 21.10% in 2017 had at least this amount of sports expenditure. The proportion of such people in the top decile is outstanding (33.40% in 2005, 27.10% in 2009, 37.10% in 2013 and 42.70% in 2017). Moving downwards, this share declines continuously. Apart from 2017, this share did not even reach 5% in the bottom half of the population. The proportion of people with such an amount of sports spending is clearly higher in every decile in 2017 compared to the other three years (Figure 7).

### 3.4. Inequality Indices

We have used inequality indicators based on the aggregate incomes and sports expenditure of each decile. The values of the normalized Herfindahl-Hirschmann and Gini indices can range from 0 to 1, taking a value of 0 for a completely equal distribution and a value of 1 for perfect concentration. The Q10 indicator represents the ratio of the top decile to the bottom decile and Q5 represents the top quintile to the bottom quintile ratio. The Robin-Hood index shows how incomes or sports expenditure should be redistributed in each decile in order to reach a completely equal distribution. See differences and specialisms in De Maio (2007), Jenkins and Van Kerm (2011), Jenkins (2022) and Trapeznikova (2019) [42,43,44,45]. We calculated these indices for the net incomes and sports expenditures of all households, as well as the net incomes of households with sports expenditures. We used all these indices because we wanted to obtain a robustness analysis of the relationship of inequalities.

The inequality indices show upward trends. The distribution of net income among all households is the indicator that shows the lowest degree of inequality in the case of each indicator. It was found to be increasing during the period examined, but the growth rate was found to be much lower than in the case of the other two indicators.

**Figure A2:**
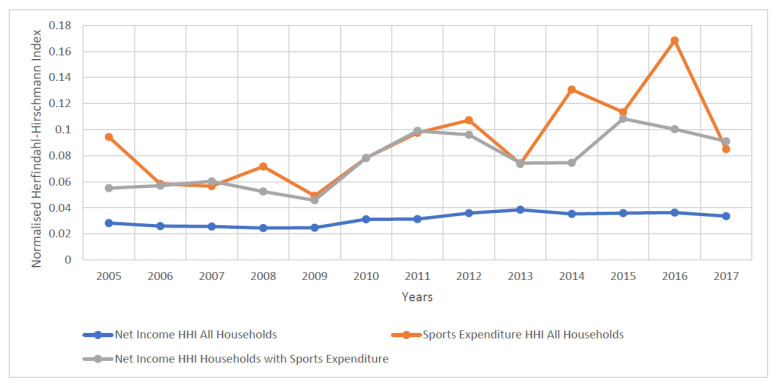
Herfinfahl Hirschmann Index (HHI) inequality indices in the time period 2005–2017. Source: own calculation.

**Figure A3:**
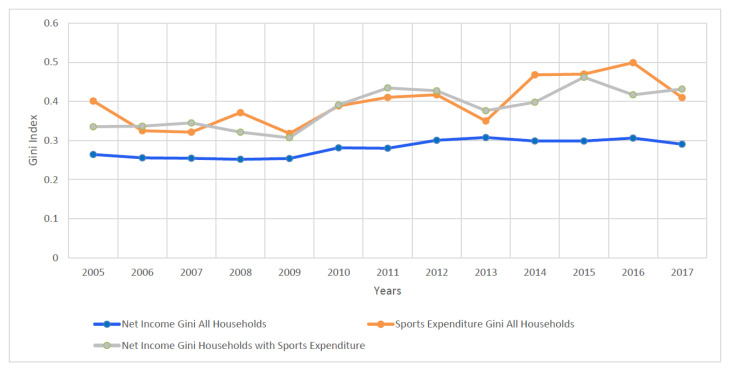
Gini inequality indices in the time period 2005–2017. Source: own calculation.

**Figure A4:**
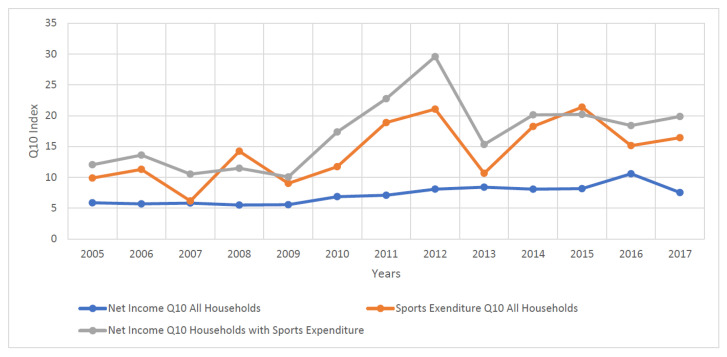
Q10 inequality indices in the time period 2005–2017. Source: own calculation.

**Figure A5:**
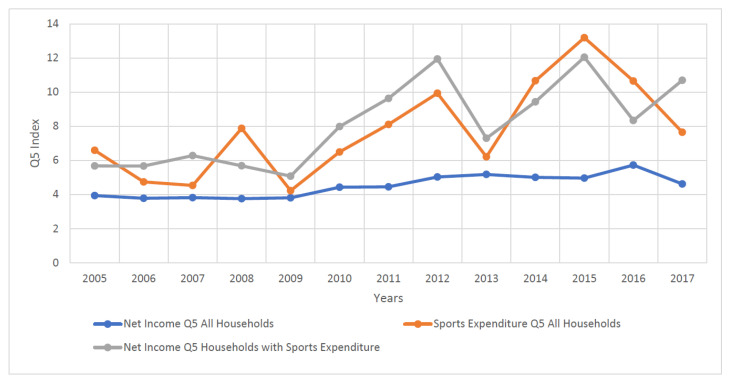
Q5 inequality indices in the time period 2005–2017. Source: own calculation.

**Figure A6:**
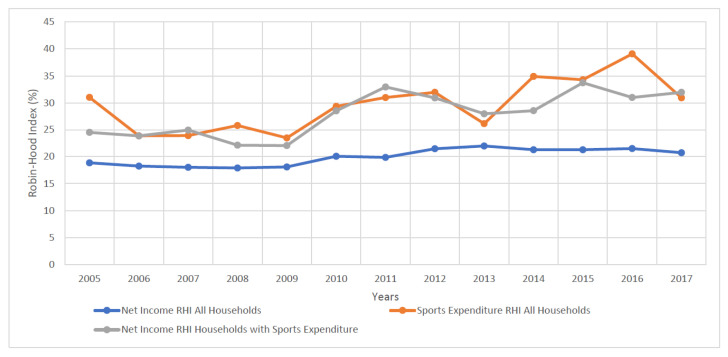
Robin-Hood inequality indices in the time period 2005–2017. Source: own calculation.

The inequalities in aggregate sports expenditures in the whole sample, as well as the inequalities in the aggregate income in the sub-sample of households with sports expenditures, show a close correlation with each other. Their growth dynamics are also very similar and show a much higher concentration than in the case of incomes in the entire sample (Table 4, Table 5 and Table 6, Appendix A, Figure A2, Figure A3, Figure A4, Figure A5 and Figure A6, Table A3, Table A4 and Table A5).

The correlation between income inequalities and sports expenditure inequalities was examined using the Pearson correlation coefficient. We compared inequality indices separately for all households and for the sub-sample of households with sports expenditures. We found a positive, moderate or strong relationship below the 5% significance level, for the period 2005–2017. Only the Q10 for all households was not significantly below the 5% significance level. The correlations show that the increase in income inequality is accompanied by an increase in inequalities in sports spending (Table 7).

## 4. Discussion

Several studies have shown that, the higher an individual’s income, the more likely they are to spend on sports, whether active or passive [41,46,47]. In addition, some studies have argued that sports can be seen as a luxury good [39,40,48]. For Hungary, a cross-sectional analysis of sports expenditure by income quintiles has already been carried out [39], but no cross-sectional or time-series analysis for income deciles.

Based on the results of the present research, there are two distinct phases in the income trend of Hungarian households in the period examined: aggregate income declined between 2005 and 2012 and then started to increase again between 2013 and 2017. The same dichotomy can be observed at the level of deciles, except for the top and bottom deciles. The top decile started to see an increase in real income by 2009, much earlier than the other deciles, while the bottom decile only started to reverse the trend by 2016. The top income decile returned to the 2005 base income level by 2013, while the other income deciles reached that level by 2015–2017. At the same time, the bottom income decile still had not returned to 2005 real income levels by 2017.

Deciles 8, 9 and 10 were able to perform above the average real income level of the general population, while the average income of all other deciles was below this level during the period under review. Narrowing down the sample to households with sport expenditures in each year, average per capita incomes were found to be higher for this sub-sample relative to the entire population. However, significant differences between the complete sample and the sub-sample with sport expenditures were found only for the bottom and top income deciles, typically in favor of households spending on sports.

The bottom and top income deciles diverged upwards and downwards respectively in income relative to the other deciles. The deterioration in living standards affected the bottom income deciles for much longer and to a greater extent than the top deciles. All the income inequality indicators show increasing income concentration between 2005 and 2017, which confirms the polarization of income in Hungary [49,50]. This suggests that wealthy households were in a more favourable position in terms of sports expenditure during the researched period.

Aggregate sports expenditure on “equipment for sports, camping and open-air recreation”, as well as “fees for sports and recreational courses”, showed an increase between 2005 and 2014. The first one doubled, although its share in total sports expenditure remained between 7–11%. The second one increased by around 27%, but in absolute terms this represents an increase of more than 7 billion HUF, as it is also the largest share of sport expenditure, at between 54% and 61%. These two items are also those most directly related to participation in active sport, which can be compared with Eurobarometer data (see later) [32,33,34].

Averages of sports expenditure per capita show a steady increase towards the upper-income deciles, accelerating from income deciles 6 and 7. As was observed for real per capita income, the 9th and 10th deciles are those with average sports expenditure values higher than the averages of the total samples for each year.

The proportion of people with sports expenditures in the total population fluctuated between 20.24% and 26.63%. This proportion, much like the income situation, showed a downward trend until 2007 and an upward trend from 2007 onwards.

In the years examined, 2005, 2009 and 2013, there was a particularly low proportion of people with sports expenditures in the bottom income decile, roughly an average for deciles 2–9, but the top decile showed an outstanding proportion. This shows that, apart from the two bottom deciles, sports played a part in the lives of almost the same proportion for most of the population in these years. By contrast, there was a more pronounced polarization in 2017, as the first three deciles showed a significant gap compared to the others, while rates in the top two deciles were at the same level as in the other three years, even though all the other deciles had worse rates in this particular year.

As about a quarter or a fifth of the Hungarian population had sports expenditures, there was a significant difference between the total sample and the sub-sample with sports expenditures, in real terms of sports expenditures for each year.

The proportion of people with sports expenditures implies that the top two deciles continued to be in a better situation than the average of the whole sample, both in the case of income and sports expenditure.

Inequalities in sports expenditure within the population have shown an increasing trend for all inequality indicators in the observed period, although some fluctuations can be found in the data. The correlation of sports expenditure and income inequality indicators confirms that income conditions move together significantly with the level of sports expenditure and that income polarization also correlates to polarization in sports expenditure.

**Figure 8:**
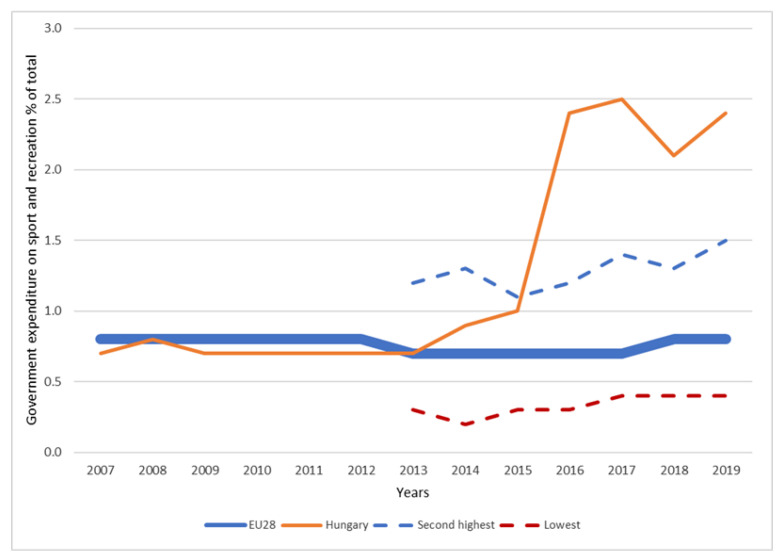
Governmental sport expenditure as a proportion of the GDP in the EU28, in Hungary and in the countries with the second highest and lowest values [51].

Hungary has been spending a significant share of its central budget on sports as a proportion of GDP in a European comparison since 2014 (Figure 8). In addition, the redirection of government corporate tax revenues into the sports sector has also led to a significant inflow of resources since 2011. The increasing trend in government and household sports expenditures can be compared with active and passive sports consumption data.

The proportion of people participating in active sports at least once per week in the EU stagnated between 2009 and 2017. An improvement could be observed in Hungary between 2009 and 2013 but this indicator has tended to stagnate since then [32,33,34]. Financial reasons as a barrier to participation in sports were highly significant in public opinion surveys in 2013 and 2017 [32,34]. Data from the EU SILC [31] database indicate that there are much larger differences in the willingness to participate in sports among the income quintiles in Hungary than the EU average. This is in line with the findings of Paár [39], Paár et al. [52] and Laczkó et al. [53] that higher income and social status also lead to higher sports expenditure and a higher likelihood of sports participation. At the same time, the sporting activity of a country’s inhabitants depends not only on socio-economic determinants but also on their (sports)cultural background and habits. Therefore, a significant increase in public spending on sports alone does not necessarily lead to an immediate improvement in sports participation and spending. This can only be hoped for if the focus of sports policy goes beyond improving access to encouraging a change in public attitudes.

While time series data are not available for passive sports consumption, cross-sectional data from 2015 shows that Hungary is slightly worse than the European average, while the related aggregate sports expenditure items show stagnation in our study period.

The growing income inequality in Hungary can be seen as a natural consequence of the capitalist market economy, as income inequality indicators have been steadily increasing in the former socialist bloc countries, but still lag vis-à-vis the traditional capitalist countries. Western European countries not only have higher living standards (life expectancy, disposable income, sports expenditure, etc.), but also higher income inequality [27,35,36].

Increasing income inequality is undesirable for several reasons: (1) it can lead to a deterioration in subjective health through the subjective perception of the relative social position of individuals [54], (2) it leads to inequalities in consumption [21], and (3) it undermines social stability [2,19]. (4) Increasing income inequalities may also prevent access to sporting opportunities for broader segments of society [38]. The observed increase in inequalities in sports consumption in the current research is also associated with an increase in income inequality. This can be assessed as a negative trend, as it can negatively affect several health indicators through multiple transmission mechanisms [24,25,26].

The main limitation of the current research is that the sports expenditure data collected by the HCSO based on COICOP categories do not correspond exactly to active and passive sports consumption activities. A more detailed breakdown of these categories would be desirable in the future. It is also difficult to carry out a full analysis of sports expenditure because a number of items related to either active (sports clothing, sports footwear, expenditure related to school sport, etc.) or passive (consumption of sports media, etc.) sports consumption cannot be precisely identified, or are included in other main categories of the COICOP classification, together with other irrelevant items. These could not be objectively included in the analysis.

An important limitation which restricts the comparison of expenditure data during the whole examined period is the methodological change in the data collection in 2015. 

It would also provide valuable insights if income and sports expenditure data on inequalities could be compared with time spent on sports consumption, but the HBS does not collect time-use data.

## 5. Conclusions

The polarization of Hungarian society during the observed period was continuous, which is also reflected in income and sports consumption. The income trend, as one of the most important determinants of access to sports, is shifting private sector spending on sports towards wealthy households to an increasing extent, while sports expenditure in the private sector is steadily increasing overall [55,56]. This means that we are talking about an expanding sector, where most of the solvent market demand is constituted by wealthy households.

This justifies the increased public or civil sector involvement in order to make sports to become more accessible to the deprived part of society [57,58]. This cause can be supported by supporting access to sports facilities (e.g., facility development, operation of community sports facilities at affordable prices, etc.), by ensuring access to sports services (e.g., supporting extra wage income perks that encourage sports activities). Further research is needed on the crowding-out effect of redirected corporate tax subsidies on household sports expenditures, investigating whether it is able to stimulate market demand and improve sports opportunities for disadvantaged groups. A further subject of future research could be how households excluded from sports consumption due to growing income inequalities substitute their leisure consumption, either in terms of leisure time or related expenditure.

## Figures and Tables

**Table 1:** Income and sports expenditure items based on COICOP nomenclature and related price indices.

Item	Content	Used CPI
Household net income	disposable income after tax	consumer price index
Equipment expenditure for sports, camping and open-air recreation	sports equipment, sports shoes, sports hunting equipment, fishing equipment, beach equipment, camping equipment	price index of sports equipment and camping goods
Entrance fees for sports and recreation services, rental fees for sports and recreational facilities and equipment	entrance fees for sports facilities (e.g., stadiums) and sports fields, swimming pools, fitness centres, gyms	price index for services related to sports and recreational activities
Fees for sport and recreational courses	fees for out-of-school sports and recreational activities, fees for personal trainers	
Fees for other recreational services	rental of sports equipment, sports shoes, tour guide fees, playground and other amusement park services	

**Table 2:** Average net income per capita in the total sample and in the sub-sample of households with sports expenditure (HUF).

Deciles	2005		2009		2013		2017	
	Total Sample	Households with Sport Expenditure	Total Sample	Households with Sport Expenditure	Total Sample	Households with Sport Expenditure	Total Sample	Households with Sport Expenditure
1	461,207	461,403	416,844	435,124	308,948	329,069	384,870	312,509
2	658,782	659,936	599,986	602,766	505,896	506,599	664,680	666,229
3	790,828	791,062	730,884	727,456	644,121	649,198	793,139	797,001
4	901,534	901,182	843,118	839,708	760,474	755,549	935,802	931,628
5	1,009,035	1,010,432	950,386	949,368	875,235	880,136	1,074,721	1,064,373
6	1,116,542	1,110,371	1,055,332	1,054,035	992,463	992,731	1,228,880	1,231,639
7	1,242,258	1,243,539	1,172,613	1,174,703	1,129,692	1,134,425	1,374,202	1,372,222
8	1,421,567	1,429,124	1,322,266	1,313,851	1,301,737	1,305,338	1,595,671	1,608,147
9	1,705,084	1,709,852	1,564,252	1,567,783	1,597,887	1,619,388	1,884,106	1,879,639
10	2,737,654	2,855,616	2,334,908	2,520,697	2,605,589	2,837,805	3,159,514	3,389,357
Total	1,319,857	1,451,309	1,217,279	1,329,565	1,216,053	1,403,191	1,439,240	1,743,109

Source: own calculation.

**Table 3:** Average per capita sports expenditure in the total sample and in the sub-sample of households with sports expenditure (HUF).

Deciles	2005		2009		2013		2017	
	Total Sample	Households with Sport Expenditure	Total Sample	Households with Sport Expenditure	Total Sample	Households with Sport Expenditure	Total Sample	Households with Sport Expenditure
1	1585	11,245	1426	9711	1598	11,126	1129	10,505
2	1750	8665	2702	14,645	1914	9956	2024	23,045
3	1545	9643	2692	12,783	2821	14,783	1910	18,506
4	2154	12,375	2283	13,854	2802	16,495	2629	22,074
5	2432	14,160	2773	16,094	2873	18,120	2957	29,923
6	2573	15,613	2377	16,286	3449	25,968	3063	26,634
7	2702	19,130	3964	27,610	3318	23,634	5989	49,482
8	4268	26,699	4107	27,077	4330	29,921	4410	35,025
9	6550	32,042	5096	30,701	5592	29,123	10,345	59,819
10	16,601	63,170	14,488	55,993	20,327	72,716	21,156	88,336
Total	4859	26,767	4826	27,290	5816	32,626	6428	47,456

Source: own calculation.

**Table 4:** Changes in net income inequality distribution indices between 2005 and 2017, all households.

Years	Herfindahl-Hirschmann	Gini	Q10	Q5	Robin-Hood
2005	0.028	0.264	5.881	3.941	18.863
2006	0.026	0.256	5.710	3.781	18.251
2007	0.026	0.255	5.831	3.819	18.016
2008	0.024	0.252	5.515	3.761	17.906
2009	0.025	0.254	5.578	3.808	18.094
2010	0.031	0.281	6.874	4.430	20.074
2011	0.031	0.280	7.099	4.454	19.869
2012	0.036	0.301	8.094	5.032	21.472
2013	0.039	0.308	8.410	5.180	21.983
2014	0.035	0.299	8.089	5.010	21.289
2015	0.036	0.299	8.169	4.965	21.284
2016	0.036	0.306	10.588	5.725	21.508
2017	0.034	0.291	7.545	4.623	20.736

Source: own calculation.

**Table 5:** Changes in net income inequality distribution indices between 2005 and 2017, households with sports expenditures only.

Years	Herfindahl-Hirschmann	Gini	Q10	Q5	Robin-Hood
2005	0.055	0.335	12.047	5.679	24.510
2006	0.057	0.337	13.613	5.675	23.856
2007	0.060	0.345	10.527	6.283	24.933
2008	0.052	0.321	11.488	5.687	22.134
2009	0.046	0.307	10.063	5.076	22.062
2010	0.078	0.391	17.360	7.983	28.504
2011	0.099	0.434	22.761	9.636	32.948
2012	0.096	0.427	29.578	11.944	30.914
2013	0.074	0.376	15.338	7.300	27.962
2014	0.075	0.398	20.132	9.436	28.542
2015	0.108	0.462	20.218	12.047	33.725
2016	0.100	0.417	18.405	8.343	30.997
2017	0.091	0.432	19.878	10.700	31.951

Source: own calculation.

**Table 6:** Changes in sports expenditure inequality indices between 2005 and 2017, all households.

Years	Herfindahl-Hirschmann	Gini	Q10	Q5	Robin-Hood
2005	0.094	0.401	9.901	6.596	31.011
2006	0.058	0.325	11.300	4.743	23.881
2007	0.057	0.321	6.181	4.534	23.916
2008	0.072	0.371	14.239	7.882	25.791
2009	0.049	0.318	9.025	4.228	23.476
2010	0.078	0.388	11.730	6.497	29.338
2011	0.098	0.410	18.896	8.111	30.982
2012	0.107	0.417	21.067	9.939	31.950
2013	0.074	0.350	10.665	6.212	26.130
2014	0.131	0.468	18.261	10.668	34.904
2015	0.113	0.470	21.390	13.196	34.284
2016	0.168	0.499	15.149	10.654	39.089
2017	0.085	0.410	16.436	7.650	30.936

Source: own calculation.

**Table 7:** The correlations between household net income inequality indices and sports expenditure.

	All Households		Households with Sports Expenditure	
	Pearson Correlation Coefficient	*p* Value	Pearson Correlation Coefficient	*p* Value
Herfindahl-Hirschmann Indices	0.678	0.011	0.684	0.010
Gini Indices	0.718	0.006	0.743	0.004
Q10 Indices	0.530	0.063	0.860	<0.001
Q5 Indices	0.713	0.006	0.782	0.002
Robin-Hood Indices	0.714	0.006	0.728	0.005

Source: own calculation.

## Data Availability

Restrictions apply to the availability of these data. Data was obtained from Hungarian Statistical Office and are available at https://www.ksh.hu/data_request_data_claim_fulfilment (accessed on 21 June 2022) with the permission of Hungarian Statistical Office.

## Appendix A

**Table A1:** Sample sizes of HBS’s in single years.

Year	Sample Size
2005	9058
2006	8973
2007	8548
2008	7650
2009	7925
2010	9935
2011	10,041
2012	9054
2013	8106
2014	6869
2015	7180
2016	7485
2017	6959

Source: own source based on HBS’s.

**Table A2:** Aggregate values of sports expenditure and income between 2005 and 2017 (million HUF).

Year	Equipment for Sports, Camping and Open-Air Recreation	Entrance Fees for Sports and Recreation Services, Rental Fee of Sports and Recreational Facilities and Equipment	Fees for Sports and Recreational Courses	Fees for Other Recreational Services	Total Sports Expenditure	Net Income
2005	3199	14,637	25,999	4525	48,360	11,898,500
2006	3768	11,732	28,755	5027	49,282	11,963,880
2007	3324	13,091	24,330	3309	44,053	11,520,337
2008	3568	13,294	26,491	3771	47,123	11,365,550
2009	3880	14,144	24,683	2663	45,371	10,803,229
2010	4538	13,149	24,470	3454	45,612	11,009,219
2011	4612	11,402	26,748	4137	46,899	11,119,718
2012	4866	13,493	31,777	3354	53,491	10,101,963
2013	4749	11,773	30,876	3561	50,959	10,449,748
2014	6520	14,261	33,208	3044	57,033	10,976,783
2015	6491	14,366	50,676	403	71,937	11,530,090
2016	6921	15,405	59,854	854	83,033	11,443,047
2017	14,757	18,005	23,154	1364	57,280	12,465,316

Source: own calculation.

**Table A3:** Correlation (and *p*-value) of net income inequality distribution indices between 2005 and 2017, all households.

	HHI	Gini	Q10	Q5	Robin-Hood
HHI	-	0.995 (<0.001)	0.894 (<0.001)	0.947 (<0.001)	0.996 (<0.001)
Gini	0.995 (<0.001)	-	0.922 (<0.001)	0.969 (<0.001)	0.998 (<0.001)
Q10	0.894 (<0.001)	0.922 (<0.001)	-	0.986 (<0.001)	0.897 (<0.001)
Q5	0.947 (<0.001)	0.969 (<0.001)	0.986 (<0.001)	-	0.952 (<0.001)
RH	0.996 (<0.001)	0.998 (<0.001)	0.897 (<0.001)	0.952 (<0.001)	-

Source: own calculation.

**Table A4:** Correlation (and *p*-value) of net income inequality distribution indices between 2005 and 2017, households with sports expenditures only.

	HHI	Gini	Q10	Q5	RH
HHI	-	0.980 (<0.001)	0.825 (0.001)	0.901 (<0.001)	0.979 (<0.001)
Gini	0.979 (<0.001)	-	0.845 (<0.001)	0.948 (<0.001)	0.992 (<0.001)
Q10	0.825 (0.001)	0.845 (<0.001)	-	0.902 (<0.001)	0.820 (0.001)
Q5	0.901 (<0.001)	0.948 (<0.001)	0.902 (<0.001)	-	0.913 (<0.001)
RH	0.979 (<0.001)	0.992 (<0.001)	0.820 (0.001)	0.913 (<0.001)	-

Source: own calculation.

**Table A5:** Correlation (and *p*-value) of sports expenditure inequality indices between 2005 and 2017, all households.

	HHI	Gini	Q10	Q5	RH
HHI	-	0.953 (<0.001)	0.617 (0.025)	0.824 (0.001)	0.970 (<0.001)
Gini	0.953 (<0.001)	-	0.742 (0.004)	0.917 (<0.001)	0.985 (<0.001)
Q10	0.617 (0.025)	0.742 (0.004)	-	0.867 (<0.001)	0.680 (0.011)
Q5	0.824 (0.001)	0.917 (<0.001)	0.867 (<0.001)	-	0.854 (<0.001)
RH	0.970 (<0.001)	0.985 (<0.001)	0.680 (0.011)	0.854 (<0.001)	-

Source: own calculation.
